# HIV Aspartic Peptidase Inhibitors Modulate Surface Molecules and Enzyme Activities Involved with Physiopathological Events in *Fonsecaea pedrosoi*

**DOI:** 10.3389/fmicb.2017.00918

**Published:** 2017-05-19

**Authors:** Vanila F. Palmeira, Daniela S. Alviano, Lys A. Braga-Silva, Fátima R. V. Goulart, Marcela Q. Granato, Sonia Rozental, Celuta S. Alviano, André L. S. Santos, Lucimar F. Kneipp

**Affiliations:** ^1^Laboratório de Investigação de Peptidases, Departamento de Microbiologia Geral, Instituto de Microbiologia Paulo de Góes, Universidade Federal do Rio de JaneiroRio de Janeiro, Brazil; ^2^Laboratório de Estrutura de Microrganismos, Departamento de Microbiologia Geral, Universidade Federal do Rio de JaneiroRio de Janeiro, Brazil; ^3^Programa de Pós-Graduação em Bioquímica, Instituto de Química, Universidade Federal do Rio de JaneiroRio de Janeiro, Brazil; ^4^Laboratório de Taxonomia, Bioquímica e Bioprospecção de Fungos, Instituto Oswaldo Cruz, Fundação Oswaldo CruzRio de Janeiro, Brazil; ^5^Laboratório de Biologia Celular de Fungos, Instituto de Biofísica Carlos Chagas Filho, Universidade Federal do Rio de JaneiroRio de Janeiro, Brazil

**Keywords:** chromoblastomycosis, *Fonsecaea pedrosoi*, peptidases, HIV aspartic peptidase inhibitors, antifungal action

## Abstract

*Fonsecaea pedrosoi* is the main etiological agent of chromoblastomycosis, a recalcitrant disease that is extremely difficult to treat. Therefore, new chemotherapeutics to combat this fungal infection are urgently needed. Although aspartic peptidase inhibitors (PIs) currently used in the treatment of human immunodeficiency virus (HIV) have shown anti-*F. pedrosoi* activity their exact mechanisms of action have not been elucidated. In the present study, we have investigated the effects of four HIV-PIs on crucial virulence attributes expressed by *F. pedrosoi* conidial cells, including surface molecules and secreted enzymes, both of which are directly involved in the disease development. In all the experiments, conidia were treated with indinavir, nelfinavir, ritonavir and saquinavir (100 μM) for 24 h, and then fungal cells were used to evaluate the effects of HIV-PIs on different virulence attributes expressed by *F. pedrosoi*. In comparison to untreated controls, exposure of *F. pedrosoi* cells to HIV-PIs caused (i) reduction on the conidial granularity; (ii) irreversible surface ultrastructural alterations, such as shedding of electron dense and amorphous material from the cell wall, undulations/invaginations of the plasma membrane with and withdrawal of this membrane from the cell wall; (iii) a decrease in both mannose-rich glycoconjugates and melanin molecules and an increase in glucosylceramides on the conidial surface; (iv) inhibition of ergosterol and lanosterol production; (v) reduction in the secretion of aspartic peptidase, esterase and phospholipase; (vi) significant reduction in the viability of non-pigmented conidia compared to pigmented ones. In summary, HIV-PIs are efficient drugs with an ability to block crucial biological processes of *F. pedrosoi* and can be seriously considered as potential compounds for the development of new chromoblastomycosis chemotherapeutics.

## Introduction

*Fonsecaea pedrosoi* is a saprophyte black fungus that is the principal etiological agent of chromoblastomycosis in humans (Santos et al., 2007). This mycosis is a chronic granulomatous infection usually observed in the epidermis, dermis and subcutaneous tissue, which occurs in humid tropical and subtropical regions around the world and with high incidence in Brazil, Mexico, Venezuela, Madagascar and Japan (Rippon, 1988; Deng et al., 2015). The management of the diseases caused by *F. pedrosoi* continues to be an arduous challenge and treatment is quite dependent on an early diagnosis. However, this tends to be quite a difficult task since affected individuals are generally low-income workers engaged in agricultural or manual labor and who do not seek help before the infection becomes uncomfortable (Santos et al., 2007). The most common treatment strategy against chromoblastomycosis focuses on the use of systemic antifungal agents in conjunction with other therapies, such as the surgical removal of lesions and cryotherapy (Queiroz-Telles and Santos, 2013). The suggested drug interventions are expensive, involving high doses of itraconazole and/or terbinafine daily for over 1 year. Even under such treatment, relapses are very common (Santos et al., 2007; Queiroz-Telles and Santos, 2013). Although some antifungals are available for treating chromoblastomycosis they act on relatively few distinct molecular targets and the emergence of resistance is a frequent problem (Andrade et al., 2004). Thus, the search for new targets and novel therapeutic strategies are the primary challenges in the sustained effort to combat this debilitating mycosis.

Proteolytic enzymes are well-known virulence factors produced by numerous opportunistic/pathogenic human fungi (Santos, 2011a). Aspartic-type peptidases, in particular, participate in essential metabolic events of a fungal cell, including nutrition, growth, proliferation, differentiation, signaling and regulated death pathways. Aspartic peptidases also help fungi during distinct facets of the interaction with the host, including (i) degradation of extracellular matrix components for dissemination, (ii) adhesion to host structures, (iii) invasion and evasion of host cells, and (iv) immune escape by the cleavage of proteinaceous components from the host response arsenal which includes immunoglobulins, complement system proteins, interleukins and antimicrobial peptides (Monod et al., 2002; Naglik et al., 2003; Santos, 2011b). Therefore, aspartic peptidases have emerged as potential targets to the development of new antifungal chemotherapeutics. Corroborating these findings, several groups have reported that aspartic peptidase inhibitors (PIs) used in anti-human immunodeficiency virus (HIV) therapy (e.g., nelfinavir, indinavir, saquinavir, ritonavir, tipranavir, amprenavir, and lopinavir) delivered antifungal effects *in vitro* and *in vivo* (Borg-von Zepelin et al., 1999; Cassone et al., 1999; Monari et al., 2005; Cenci et al., 2008; Santos et al., 2013). For instance, the HIV-PIs ritonavir and indinavir demonstrated anti-*Candida albicans* activity in a rat vaginitis model (Cassone et al., 1999). In *Cryptococcus neoformans*, tipranavir showed a therapeutic effect in experimental systemic cryptococcosis, reducing fungal burden in the brain and the liver of both immunocompetent and immunodepressed mice (Cenci et al., 2008).

Our research group previously demonstrated that both the conidial and the mycelial forms of *F. pedrosoi* secreted aspartic peptidases into the extracellular environment and that these enzymes were able to degrade extracellular matrix-forming proteins (laminin, fibronectin and collagen) as well as serum proteins (albumin, IgG and fibrinogen) (Palmeira et al., 2006a,b). Interestingly, clinical strains of *F. pedrosoi*, freshly isolated from human lesions, produced higher levels of extracellular aspartic peptidase activity compared to a long time laboratory-adapted strain, suggesting that enzyme production may be stimulated by interaction with the host (Palmeira et al., 2006b). Furthermore, we demonstrated that nelfinavir, saquinavir, ritonavir and indinavir inhibited the secreted aspartic proteolytic activity of conidia and mycelia of *F. pedrosoi*, in a dose-dependent manner (Palmeira et al., 2006b, 2008). Drastic morphological alterations were also observed after treatment of *F. pedrosoi* with HIV-PIs, which culminated in conidial death. These morphological perturbations led to an inability of conidia to (i) adhere to and enter into animal cells, (ii) differentiate into mycelia, and (iii) resist macrophage killing mechanisms (Palmeira et al., 2008).

Herein, we report the alterations in the production of relevant and crucial biomolecules by *F. pedrosoi* conidia upon treatment with HIV-PIs. In this context, we have focused on surface molecules (mannose- and sialic acid-containing glycoconjugates, glucosylceramide, melanin and sterol) and extracellular hydrolytic enzymes (peptidase, esterase and phospholipase), which act as potential virulence attributes of this human opportunistic fungus (Soares et al., 1995; Limongi et al., 1997; Alviano et al., 2004a,b; Nimrichter et al., 2005; Palmeira et al., 2010).

## Materials and Methods

### Chemicals

Indinavir, saquinavir, ritonavir and nelfinavir were obtained from the National Institutes of Health (NIH), and were dissolved in methanol to obtain a final concentration of 20 mM. Bovine serum albumin (BSA), pepstatin A, Tween 80, ergosterol, lanosterol, fluorescein isothiocyanate (FITC)-labeled secondary antibodies, and FITC-Concanavalin A (Con A) and FITC-*Sambucus nigra* (SNA) agglutinins were purchased from Sigma-Aldrich (St. Louis, MO, United States). Media constituents, reagents used in microscopy and buffer components were purchased from Amersham (Little Chalfont, United Kingdom). All other reagents were of analytical grade.

### Microorganism and Growth Conditions

A pathogenic strain of *F. pedrosoi* (ATCC 46428, formerly 5VLP), isolated from a human patient with chromoblastomycosis (Oliveira et al., 1973), was used throughout the experimental work. For conidium formation, cultures were incubated for 5 days under constant agitation (200 rpm) at room temperature in 250-ml Erlenmeyer flasks containing 100 ml of Czapek-Dox chemically defined medium (Palmeira et al., 2008). The fungal cultures were centrifuged (4000 × *g*, 10 min, 4°C) and the conidia were washed three times in phosphate-buffered saline (PBS; 150 mM NaCl, 20 mM phosphate buffer, pH 7.2). Growth was estimated by counting the conidia in a Neubauer chamber.

### Fungal Treatment with HIV-PIs

*Fonsecaea pedrosoi* conidia (10^3^ or 10^6^ cells) were incubated with each HIV-PI (indinavir, saquinavir, ritonavir and nelfinavir) at 100 μM concentration for 24 h at room temperature. Afterward, conidia were washed three times in PBS and viability was assessed by colony-forming unit (CFU) measurements (Palmeira et al., 2008). The untreated and methanol-treated conidia were used in all the experimental sets. For all the subsequent experiments (except when discriminated), 10^6^ conidia were treated (or not) with each HIV-PI at 100 μM for 24 h in order to detect (i) morphological changes, (ii) expression of surface molecules, and (iii) extracellular enzymes.

### Morphological Parameters

Conidia were processed for flow cytometry in order to measure two morphological parameters: size and granularity. Briefly, conidia were centrifuged (4000 × *g*, 10 min, 4°C), washed with PBS and fixed in 4% paraformaldehyde at 4°C for 30 min. Then, fungal cells were analyzed in a flow cytometer (FACSCalibur; BD Bioscience) equipped with a 15-mW argon laser emitting at 488 nm. Each experimental population was mapped (*n* = 10,000 events) using a two parameter histogram of forward-angle light scatter (FSC) versus side scatter (SSC), respectively, to evaluate cellular size and granularity (Braga-Silva and Santos, 2011).

### Ultrastructural Analysis

Conidia were fixed with a solution containing 2.5% glutaraldehyde, 4% paraformaldehyde, 10 mM CaCl_2_ in 0.1 M cacodylate buffer (pH 7.2) for 1 h at 25°C. Then, conidia were washed with the same buffer and post-fixed for 1 h in a solution containing 1% OsO_4_ and 0.8% potassium ferricyanide in cacodylate buffer. Subsequently, the cells were rinsed, dehydrated in a graded series of acetone and embedded in Spurr resin. Ultrathin sections were obtained, stained with uranyl acetate and lead citrate, and examined using a JEOL 1200 EX transmission electron microscope (Palmeira et al., 2008).

### Sterol Content

Conidia were subjected to successive extractions with a chloroform:methanol (2:1) mixture in order to remove lipids from the fungal cells (Soares et al., 1995). Precipitated material was removed by centrifugation and the solution was then reduced to dryness under nitrogen. Following Folch partition (Folch et al., 1957) sterol content of the lipid extract was analyzed by high-performance thin-layer chromatography (HPTLC) using as solvent mixture of hexane-ether-acetic acid (80:40:2) and revealed with a solution comprising 50 mg of FeCl_3_, 90 ml of water, 5 ml of acetic acid and 5 ml of H_2_SO_4_. The plate was heated to 100°C for 5 min and the bands of sterol were then visualized and compared to sterol standards, ergosterol and lanosterol (Larsen et al., 2004). Sterol quantitative determinations were performed using ImageJ software (Granato et al., 2017).

### Surface Molecules

For fluorocytometric analysis, conidia were fixed in 4% paraformaldehyde for 1 h, rinsed in PBS and then incubated for 1 h at room temperature in the presence of fluorescent probes, FITC-Con A (5 μg/ml) and FITC-SNA (40 μg/ml) agglutinins, in order to detect mannose- and sialic acid-containing glycoconjugates, respectively (Limongi et al., 1997; Alviano et al., 2004b). In addition, both anti-CMH and anti-melanin antibodies, at 1:100 dilution, were also incubated to detect glucosylceramide and melanin pigment, respectively (Alviano et al., 2004a; Nimrichter et al., 2005). Cells were washed and the conidia were then treated with primary antibodies followed by a 1 h incubation period with FITC-secondary antibody. In parallel, conidial cells that had not been incubated with agglutinins or antibodies were also prepared in order to run as autofluorescence control systems. Finally, fungal cells were washed in PBS and analyzed by flow cytometry. Control cells were analyzed first in order to determine their autofluorescence and relative size. Each experimental population was mapped using a two-parameter histogram of FSC versus SSC. The mapped population (*n* = 10,000 events) was analyzed for log green fluorescence by using a single-parameter histogram. The results were expressed by means of two parameters: percentage of fluorescent cells and mean of fluorescence intensity (MFI).

### Phospholipase and Esterase Assays

Determination of phospholipase production was performed by using egg-yolk agar plates (Price et al., 1982). The esterase production was assayed using Tween 80 agar medium. Briefly, conidia (10 μl containing 10^6^ cells) were placed in the center of agar plates and incubated at 37°C up to 10 days (Palmeira et al., 2010). The colony diameter (*a*) and the diameter of colony plus precipitation zone (*b*) were measured by a digital paquimeter. Phospholipase and esterase activities were expressed as P*z* value (*a*/*b*) (Price et al., 1982), in which low P*z* values mean high enzymatic production and, inversely, high P*z* values indicate low enzymatic production.

### Extracellular Proteolytic Activity

Following treatment with HIV-PIs, conidia were washed three times in PBS and re-suspended in Czapek-Dox medium for 2 h at room temperature. Subsequently, the fungal cells were harvested (4000 × *g*/10 min/4°C) and the cell-free culture supernatants were used to measure proteolytic activity (Buroker-Kilgore and Wang, 1993). Sample normalization was performed by protein concentration. The protein content was measured using the method described by Lowry et al. (1951), using BSA as standard. Briefly, 50 μl of supernatant (equivalent to 1 μg of protein), BSA (0.5 mg/ml) and 10 mM sodium citrate (pH 4.0) were added to a microcentrifuge tube (350 μl) and incubated for 1 h at 37°C. Afterward, three aliquots (100 μl each) of the reaction mixture were transferred to wells on a microtiter plate containing 50 μl of water and 100 μl of a Coomassie solution (0.025% Coomassie brilliant blue G-250, 11.75% ethanol and 21.25% phosphoric acid). A control, in which the substrate was added just after the reactions were stopped, was used as a blank. After 10 min (to allow for dye binding) the plate was read on a Molecular Devices Thermomax microplate reader at 595 nm. One unit of enzyme activity was defined as the amount of enzyme that caused an increase of 0.01 absorbance unit, under standard assay conditions (Palmeira et al., 2006b, 2008). Alternatively, the supernatant fluids were pre-incubated for 15 min at 37°C in the absence or presence of pepstatin A (1 and 10 μM) and then their ability to cleave BSA was measured.

### Aspartic Peptidase Production and Susceptibility to HIV-PIs in Pigmented and Non-pigmented Conidia

In order to obtain non-pigmented cells, conidia were grown in flasks filled with Czapek-Dox medium almost to the top (to decrease aeration) and incubated in the dark for 5 days at room temperature under constant agitation (200 rpm) (Alviano et al., 1991). Subsequently, both pigmented and non-pigmented conidia were evaluated for the production of aspartic peptidase and their susceptibility to HIV-PIs (in this last test, 10^3^ cells were used).

### Statistical Analysis

All the experiments were repeated at least three times and all the systems were performed in triplicate. Data were analyzed statistically using Student’s *t*-test using EPI-INFO 6.04 (Database and Statistics Program for Public Health) computer software. *P* values of 0.05 or less were considered statistically significant.

## Results and Discussion

### Anti-*Fonsecaea pedrosoi* Action of HIV-PIs

**FIGURE 1:**
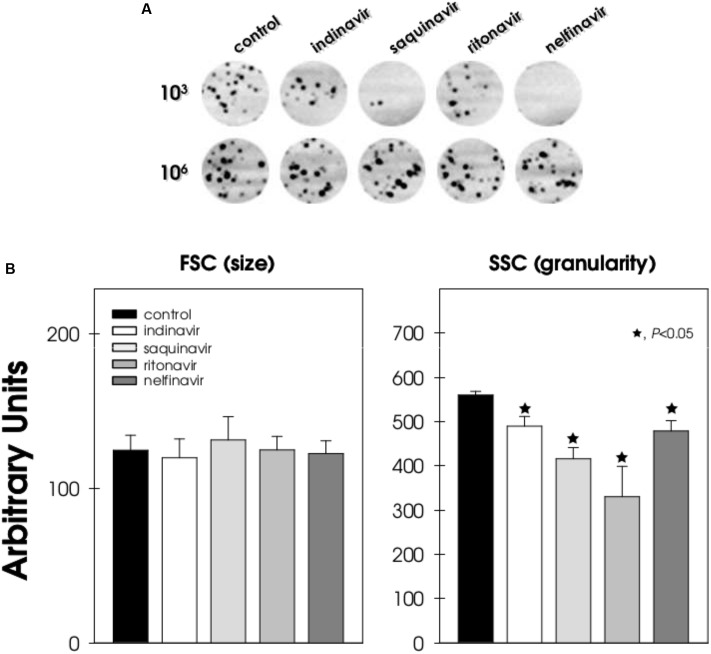
**Effect of HIV-PIs on the growth behavior and morphology of *F. pedrosoi* conidial cells. (A)** Different conidial cell densities (10^3^ and 10^6^ cells/ml) were incubated in the absence (control) or in the presence of indinavir, nelfinavir, ritonavir and saquinavir at 100 μM for 24 h. Subsequently, the mixtures were plated onto fresh solid medium without drugs. Image digitalization of the plates is shown. **(B)** In parallel, 10^6^ conidial cells/ml were treated (or not) with the HIV-PIs (100 μM) for 24 h and then analyzed by flow cytometry in order to measure cell size and granularity. Forward scatter (FSC) measurement is related to cell size and side scatter (SSC) measurement is related to the internal granularity and/or complexity of a cell. The values represent the mean ± standard deviation of three independent experiments performed in triplicate. Symbols (★72) indicate the experimental systems considered statistically significant from the control (*P <* 0.05, Student’s *t*-test).

Aspartic peptidases produced by *F. pedrosoi* cells are apparently key regulators of growth, development, morphogenesis and interaction with host cells/tissues (Palmeira et al., 2006b, 2008; Santos et al., 2007). For instance, the classical aspartic PI, pepstatin A, was able to reduce the viability of *F. pedrosoi* conidia in a manner which was dependent upon both cell number and drug concentration (Palmeira et al., 2008). Based on this observation, we initially investigated the effect of HIV-PIs on the cellular viability of *F. pedrosoi*. In this way, two conidial cell densities (10^3^ and 10^6^ cells/ml) were each treated for 24 h with 100 μM HIV-PIs, and then plated onto drug-free solid medium in order to quantify the CFU. As previously shown by our group (Palmeira et al., 2008), drug treatment of 10^3^ conidia significantly reduced the cell viability, especially nelfinavir (≈100%) and saquinavir (≈90%), compared to the non-treated cells (**Figure 1A**). In contrast, the treatment of 10^6^ conidia with the HIV-PIs did not alter the growth behavior, resulting in comparable CFU numbers (**Figure 1A**). Similarly, exposing different densities of *C. albicans* yeast cells to amprenavir at a concentration of 100 μM gave the same antifungal profile (Braga-Silva and Santos, 2011). It is well-known that the antimicrobial action of a given compound depends on the microbial inoculum, drug concentration and time of treatment (Gehrt et al., 1995). In this context, for example, increasing the number of microbial targets may exceed the capability of a given drug amount to inhibit growth of the test organism. Inoculum concentration may particularly affect antifungal drugs whose antimicrobial activity is based on an enzymatic mechanism (Gehrt et al., 1995; Palmeira et al., 2006a,b, 2008).

The direct antifungal action of HIV-PIs against other opportunistic fungi, especially *C. albicans, C. parapsilosis, C. neoformans*, and *Pneumocystis jiroveci*, has been reported previously (Atzori et al., 2000; Asencio et al., 2005; Monari et al., 2005; Santos and Braga-Silva, 2013). In parallel with these earlier studies, no alteration in cell size was observed when 10^6^ *F. pedrosoi* conidia cells were treated with HIV-PIs in comparison to untreated cells. However, a significant reduction on the granularity parameter was seen in HIV-PI-treated conidia, as revealed by flow cytometry (**Figure 1B**). In contrast, amprenavir (100 μM concentration) was previously shown to cause a significant reduction (≈30%) on the size of *C. albicans* cells compared to the non-treated yeast cells, although no differences in granularity occurred (Santos and Braga-Silva, 2013).

### Ultrastructural Alterations by HIV-PIs: Focus at the Surface of *F. pedrosoi* Conidia

**FIGURE 2:**
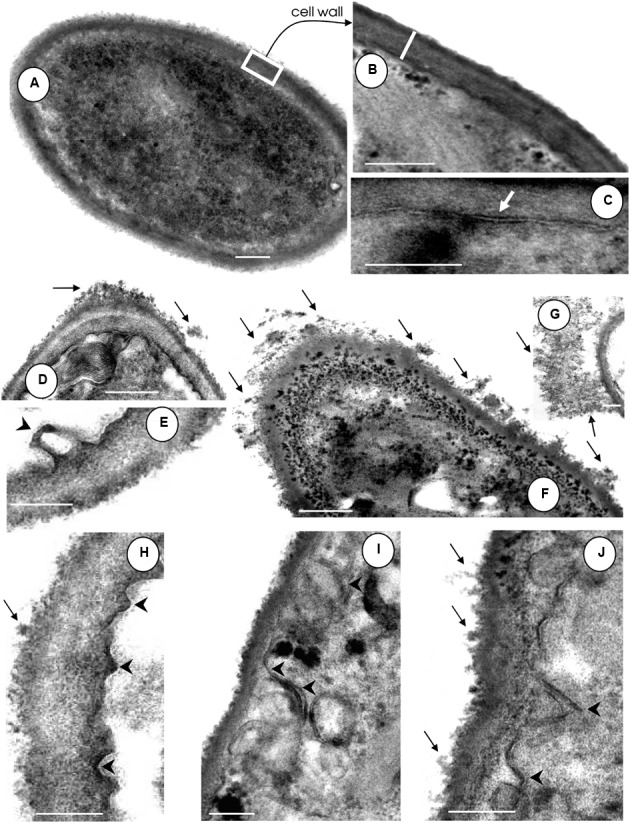
**Effect of HIV-PIs on the ultrastructure of *F. pedrosoi* conidial cells. (A–C)** Untreated and **(D–J)** HIV-PI-treated (100 μM for 24 h) conidial cells were processed and analyzed by transmission electron microscopy. Control conidial cells present a dense cytoplasm **(A)** with a distinct and compact cell wall (**B**, *white bar* shows the wall thickness) and well-delineated plasma membrane (**C**, *white arrow*). Black arrows show the releasing of electron dense and amorphous material from the cell surface and black arrowheads indicate the invaginations of the plasma membrane with consequent withdrawal from the cell wall following treatment of conidia with indinavir **(D,E)**, nelfinavir **(F,G)**, ritonavir **(H)** and saquinavir **(I,J)**. Scale bars: **(A,D,F)**, 0.4 μm; **(B–J)**, 0.25 μm.

In this set of experiments, the effects of HIV-PIs (indinavir, ritonavir and also saquinavir and nelfinavir) on the fungal cell surface were evaluated. In comparison to untreated control conidia, which had normal morphology with typical dark and dense cytoplasm (**Figure 2A**), classical cell wall (**Figure 2B**) and a well-delineated cytoplasmic membrane (**Figure 2C**), cells exposed to HIV-PIs showed significant changes in surface topography, including (i) shedding of a great amount of electron dense and amorphous material on the outer side of the cell wall (**Figures 2D,F–H,J** black arrows) and (ii) a cytoplasmic membrane presenting numerous undulations and/or invaginations as well as withdrawal of the cytoplasmic membrane from the cell wall (**Figures 2E,H,I**, arrowheads). Although all of these morphological alterations were clearly evident following treatment with the four HIV-PIs, saquinavir and nelfinavir induced the greatest effects, a result which corroborates their anti-proliferative properties and also our previous findings (Palmeira et al., 2008). Likewise, images obtained using scanning electron microscopy revealed drastic changes on the morphology of *C. albicans* yeasts treated with amprenavir (Braga-Silva et al., 2010). Surface invaginations and detachment of the external fibril layer were clearly observed, giving rise to fungal cells with a smooth surface. Additionally, the release of surface materials from the cell wall culminated in an inability of *C. albicans* to adhere and, consequently, to form a robust and mature biofilm on polystyrene (Braga-Silva et al., 2010; Braga-Silva and Santos, 2011).

### HIV-PIs Modulate the Surface Molecules Produced by *F. pedrosoi* Conidia

**FIGURE 3:**
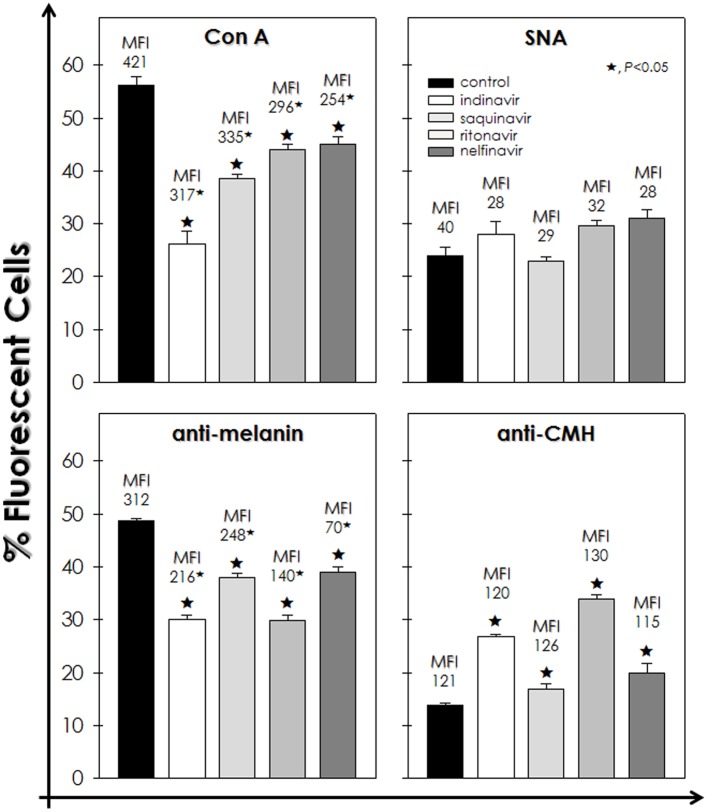
**Effect of HIV-PIs on the expression of surface molecules of *F. pedrosoi* conidial cells.** Untreated (control) and HIV-PI-treated (100 μM for 24 h) conidial cells were processed to detect mannose- and sialic acid-containing glycoconjugates by using FITC-Con A and FITC-SNA agglutinins, respectively, as well as melanin and glucosylceramide by means of anti-melanin and anti-CMH antibodies, respectively. Afterward, conidial cells were analyzed by flow cytometry and the percentages of positive-labeled cells were shown. Moreover, in each analysis, the values of mean fluorescence intensity (MFI) are shown above the bars. The autofluorescence value of conidial cells not treated with either FITC-agglutinins or antibodies was discounted from the treated ones. The values represent the mean ± standard deviation of three independent experiments performed in triplicate. Symbols (★72) indicate the experimental systems considered statistically significant from the control (*P <* 0.05, Student’s *t*-test).

Considering the observed fungal surface alterations, the effects of HIV-PIs on the expression of surface molecules (e.g., mannose- and sialic acid-containing glycoconjugates, sterol, melanin and glucosylceramide), usually expressed by *F. pedrosoi* and associated with virulence, was evaluated (**Figure 3**). In this set of experiments, 10^6^ fungal cells/ml was the cellular density selected for treatment with HIV-PIs, since at this density the conidia maintained their viability post-treatment (**Figure 1**).

Flow cytometry analyses demonstrated that indinavir, saquinavir, ritonavir and nelfinavir significantly diminished the percentage of fluorescently Con A-labeled cells by approximately 54, 32, 22, and 20%, respectively, when compared to untreated cells. In addition, it was evidenced a considerable dropped in the MFI values after the treatment with the HIV-PIs, which indicates a reduction in the surface amount of mannose-rich glycoconjugates in *F. pedrosoi* conidial cells (**Figure 3**). In contrast, the sialic acid expression was not significantly altered, since similar percentage of SNA-labeled cells and MFI values were observed in untreated and HIV-PIs-treated fungal cells (**Figure 3**). Indinavir and ritonavir diminished the number of melanin-positive cells by around 40%, while saquinavir and nelfinavir by approximately 20%. However, all the four HIV-PIs significantly reduced the MFI parameter, especially nelfinavir, suggesting a reduction on the melanin production (**Figure 3**). Interestingly, a considerable augmentation in the number of glucosylceramide-positive cells was observed after incubation with HIV-PIs as follows: ritonavir > indinavir > nelfinavir > saquinavir; however, no changes were detected in MFI values (**Figure 3**).

**FIGURE 4:**
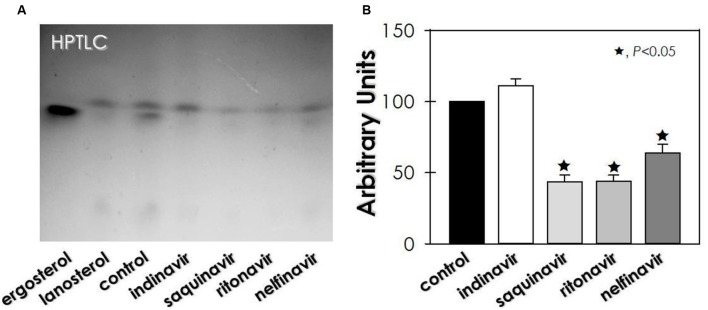
**Effect of HIV-PIs on the sterol synthesis of *F. pedrosoi* conidial cells. (A)** Representative image of untreated (control) and HIV-PI-treated (100 μM for 24 h) conidial cells. For sterol analysis, the lipid extracts were loaded into HPTLC silica plates, separated using a mixture of solvents (hexane-ether-acetic, 80:40:2) and revealed by spraying a solution containing ferric chloride, water, acetic acid and sulfuric acid, which revealed the sterol as red-violet color spots. **(B)** Densitometric analyses of the lanosterol bands, expressed in arbitrary units. Symbols (★72) indicate the experimental systems considered statistically significant from the control (*P <* 0.05, Student’s *t*-test).

Regarding sterol content, untreated conidia of *F. pedrosoi* showed an equal proportion of lanosterol (a precursor in ergosterol biosynthesis) and ergosterol (the final product) (**Figure 4**). On the other hand, no ergosterol was detected in HIV-PIs-treated conidia, whereas lanosterol production was inhibited (≈56%) by saquinavir and ritonavir, as well as by nelfinavir (≈36%) (**Figure 4**).

Adherence is one of the most important determinants of microbial pathogenesis. This step is normally followed by microbial internalization, which can be used by the pathogen to replicate inside the host cells and evade immune responses (Tronchin et al., 2008). In this way, surface-located molecules act as adhesive structures, and thus the inhibition of their synthesis and/or expression can diminish the ability of fungal cells to interact with both abiotic and biotic surfaces (Santos and Braga-Silva, 2013). Fungal mannose-containing glycoconjugates are directly involved in the initial adhesion step to host cell surface (Kohatsu et al., 2006), including in *F. pedrosoi* (Limongi et al., 1997). Our group previously demonstrated that HIV-PIs were able to diminish the adhesion and invasion capabilities of *F. pedrosoi* conidia during the interaction with epithelial cells, fibroblasts and macrophages (Palmeira et al., 2008), an effect which can now be explained, at least in part, as being due to the significant reduction in the surface level of mannose-rich glycoconjugates observed in the present study.

In several microbial species, including fungi, sialic acids are considered as anti-recognition molecules that elude the host immune system by, for example, protecting against phagocytosis (Alviano et al., 2004b). Our current study showed that the HIV-PIs treatment did not considerably alter the expression of sialic acid on surface glycoconjugates of *F. pedrosoi* conidia. Similarly, *C. albicans* cells treated with the HIV-PI, amprenavir, at the same concentration used herein (100 μM), did not modulate the expression of surface sialic acid molecules (Braga-Silva et al., 2010).

It is well-known that *F. pedrosoi* constitutively produces the pigment, melanin, which is a complex polymer that is deposited on the cell wall and in cytoplasmic structures (melanosomes) as well as being released into the extracellular environment (Alviano et al., 1991, 2004a; Rozental et al., 1994; Cunha et al., 2010). Melanin is a classical virulence factor due to its ability to protect fungal cells against oxidative stressors, to inhibit cell-mediated host immune responses, to interfere with complement cascade activation and to reduce the susceptibility to antifungal agents (Bocca et al., 2006; Nosanchuk and Casadevall, 2006; Santos et al., 2007). Moreover, melanin is able to block the access of several molecules through the cell wall (Nimrichter et al., 2005; Nosanchuk and Casadevall, 2006). For example, high melanized conidia of *F. pedrosoi* blocked the access of antibodies against glucosylceramide, an antigenic sphingolipid involved in fungal growth and differentiation (Nimrichter et al., 2005; Del Poeta et al., 2014). In contrast, anti-glucosylceramide antibody was able to efficiently penetrate the cell wall and bind to its target in melanin-depleted *F. pedrosoi* cells, culminating in fungal death (Nimrichter et al., 2005). Interestingly, indinavir and ritonavir were the most effective HIV-PIs for reducing the production of melanin, concomitantly to the increased recognition of anti-glucosylceramide antibody to the surface of *F. pedrosoi* conidial cells. Also, the reduced melanin-producing capability of HIV-PIs-treated conidia, as observed herein, helps explain the previously reported enhanced killing capacity of macrophages (Palmeira et al., 2008). Corroborating our hypothesis, the inhibition of the melanin synthesis pathway by tricyclazole also increased the susceptibility of *F. pedrosoi* conidia to mouse macrophages (Cunha et al., 2005). Furthermore, a very interesting, previously published study revealed a positive correlation between human melanogenesis and aspartic peptidase activity (Rochin et al., 2013). Briefly, the β-secretase BACE2 (beta-site cleaving enzyme), which is an aspartic-type peptidase, is able to process the pigment cell-specific protein melanocyte (PMEL) to form the melanosome amyloid matrix in pigment cells. The pharmacological inhibition of BACE2, or its RNA silencing, interfered in the melanosome morphogenesis and, consequently, in pigmentation defect as shown in *in vitro* studies using the human melanocytic MNT1 cell line as well as in *in vivo* studies using Bace2^-/-^ mice.

Almost all of the current test HIV-PIs were also able to decrease the sterol content in *F. pedrosoi* conidia. The disturbance of sterol synthesis, a bioregulator of membrane fluidity, asymmetry and integrity, may lead the fungal cells to death (Kathiravan et al., 2012). In this sense, the ultrastructural changes observed in HIV-PI-treated *F. pedrosoi* conidia [also reported by Palmeira et al. (2008)], including invaginations in the cytoplasmic membrane and detachment of this structure from the cell wall, culminated in abnormal cellular division, the disfiguring of regular conidia morphology and irreversible injuries that ultimately led to conidia death.

### Effect of HIV-PIs on the Secretion of Hydrolytic Enzymes in *F. pedrosoi* Conidia

**FIGURE 5:**
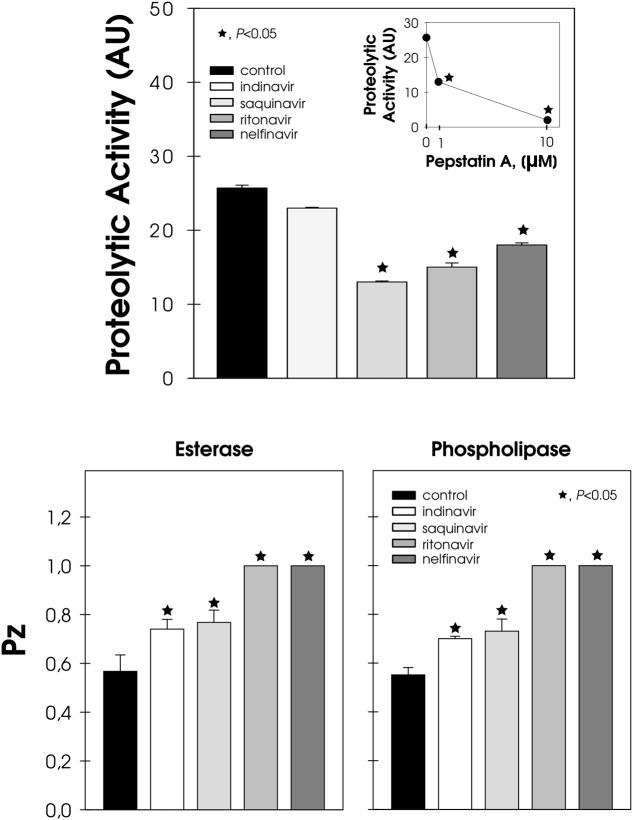
**Effect of HIV-PIs on the secretion of hydrolytic enzymes by *F. pedrosoi* conidial cells.** Untreated (control) and HIV-PI-treated (100 μM for 24 h) conidial cells were washed and re-suspended in Czapek-Dox medium for an additional 2 h. After this period, the cell-free culture supernatants were submitted to a proteolytic activity assay. In addition, the supernatant obtained from the control cells was pre-incubated in either the absence or presence of pepstatin A (1 and 10 μM) and then checked for their ability to cleave BSA (*inset graphic*). The proteolytic activities were expressed as arbitrary units (AU). In parallel, conidia treated (or not) with HIV-PI were placed in the center of either Tween 80 or egg-yolk agar plates to detect esterase and phospholipase activities, respectively. The lipase activities were expressed as Pz and the values represent the mean ± standard deviation of three independent experiments performed in triplicate. Symbols (★72) indicate the experimental systems considered statistically significant from the control (*P <* 0.05, Student’s *t*-test).

Conidia cells were able to extracellularly release aspartic-type peptidase, whose hydrolytic activity was inhibited by pepstatin A, in a typical dose-dependent manner (**Figure 5**, inset). Conversely, the pre-treatment of conidia with HIV-PIs, particularly saquinavir, ritonavir and nelfinavir, notably arrested the secretion of pepstatin A-sensitive aspartic peptidase, inhibiting this activity by around 50, 40, and 30%, respectively (**Figure 5**). HIV-PIs also interfered with the production of esterase and phospholipase, which are two unrelated enzymes belonging to the lipase class (**Figure 5**). Ritonavir and nelfinavir inhibited lipolytic activities by around 70%, while indinavir and saquinavir affected substrate hydrolysis by approximately 30% (**Figure 5**).

Peptidases and lipases are ubiquitous enzymes involved in diverse processes, which includes homeostasis, nutrient acquisition and fungal virulence (Santos, 2011a; Park et al., 2013). As fungal enzymes play roles in several crucial pathophysiological events, the inhibition of at least one of the many stages controlled by them will probably contribute to the containment of the pathogen and thus should help to control disease development (Santos, 2011a; Park et al., 2013). In this sense, HIV-PIs were able to directly inhibit the enzymatic activity of aspartic peptidase secreted by *F. pedrosoi* (Palmeira et al., 2008) and also, as demonstrated herein, its secretion into the extracellular milieu. *F. pedrosoi* is also renowned as an excellent producer of esterase and phospholipase (Palmeira et al., 2010). Interestingly, HIV-PIs have previously been shown to significantly reduce the secretion of both of the latter lipases by *C. albicans* (Cenci et al., 2008; Braga-Silva et al., 2010). The inhibitory capability of non-proteolytic enzymes by HIV-PIs was also observed during the treatment of *C. neoformans* with indinavir, with the drugs prompting a significant reduction in the production of urease, which is an essential virulence factor of this opportunistic fungus (Monari et al., 2005). Indinavir also powerfully inhibited capsule formation, another major virulence factor of *C. neoformans*, whilst both melanin and phospholipase productions were unaffected by this HIV-PI (Monari et al., 2005). Moreover, HIV-PIs were active in blocking the morphological transition, which is recognized as an integral step in the establishment of a successful fungal infection by *C. albicans* (Braga-Silva and Santos, 2011) and *F. pedrosoi* (Palmeira et al., 2008).

### Correlation among Melanin, Secretion of Aspartic Peptidase, and Susceptibility to HIV-PIs

**FIGURE 6:**
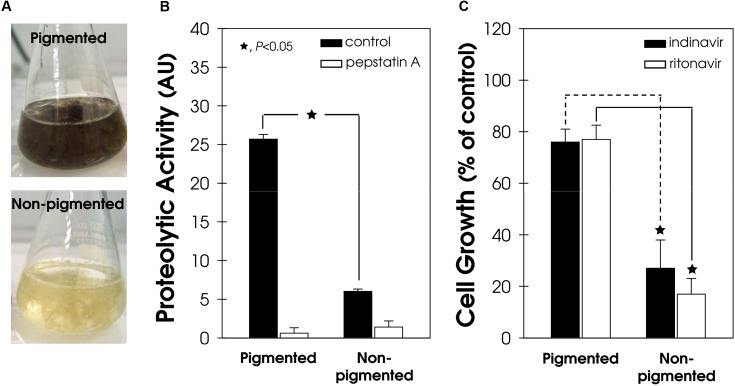
**Secretion of aspartic peptidase and susceptibility to HIV-PIs in pigmented and non-pigmented *F. pedrosoi* conidial cells. (A)** Representative image of pigmented (melanized) and non-pigmented (non-melanized) *in vitro* conidial growth. **(B)** The cell-free culture supernatants from both pigmented and non-pigmented conidia were used to quantify the acidic proteolytic activity, using BSA as the soluble protein substrate, in the absence/presence (10 μM) of pepstatin A. The proteolytic activities were expressed as arbitrary units (AU). **(C)** Conidia (10^3^ cells/ml) were incubated in the absence (control) or in the presence of indinavir and ritonavir (100 μM) for 24 h. Subsequently, the mixtures were plated on fresh solid medium without drugs. In all graphics, the values represent the mean ± standard deviation of three independent experiments performed in triplicate. Symbols (★72) indicate the experimental systems considered statistically significant from the control (*P <* 0.05, Student’s *t*-test).

The secretion of aspartic peptidase in both pigmented and non-pigmented conidial cells of *F. pedrosoi* (**Figure 6A**) was also evaluated. The aspartic-type peptidase activity was four-fold higher in pigmented conidial cells compared to the non-pigmented ones (**Figure 6B**). In the same way, the process of melanization in phytopathogenic fungi directly affects the secretion of proteins, including lytic enzymes (Henson et al., 1999). For instance, the spot blotch caused by the fungus *Bipolaris sorokiniana* is one of the most important diseases of wheat and barley in South Asia and, recently, a positive correlation was found between high melanin production, elevated amounts of extracellular enzymes, including peptidase, and the aggressiveness of isolates from naturally infected barley (Chand et al., 2014).

Melanin can reduce the fungal susceptibility to different classes of antifungal agents (Cunha et al., 2005) because it forms a protecting shield around the fungal cell. In support of this finding, indinavir and ritonavir only slightly reduced the viability of pigmented *F. pedrosoi* cells by around 23%, while non-pigmented conidia were drastically affected by exposure to both of these HIV-PIs (**Figure 6C**).

## Conclusion

**FIGURE 7:**
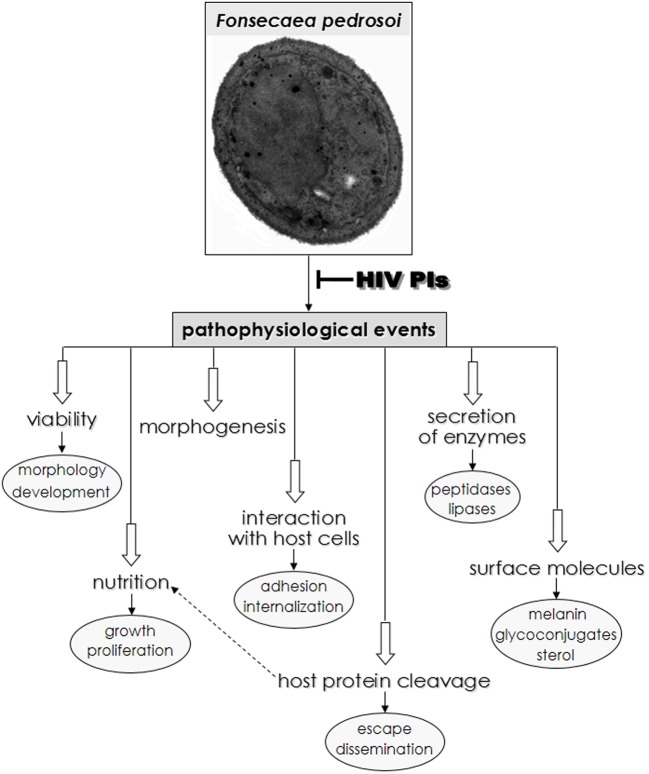
**Overview of possible HIV-PIs action on *F. pedrosoi* conidial cells in central metabolic processes as well as in distinct phases of interaction with host cell structures.** Both physiological and pathological events that occur in some species of yeast and filamentous fungi, including *F. pedrosoi* are inhibited, in different extensions, by the HIV-PIs (for review see Santos et al., 2013).

Chromoblastomycosis disease is often recalcitrant and difficult to treat using available pharmacotherapy. Taken together, our results clearly show that HIV-PIs, especially nelfinavir and saquinavir, dramatically affect *F. pedrosoi* conidia growth and ultrastructure. Moreover, HIV-PIs modulate *F. pedrosoi* sterol content and important surface molecules, including melanin and glucosylceramide, as well as secreted enzymes (aspartic peptidase, esterase and phospholipase). The results obtained in the current and previous studies (Palmeira et al., 2008) revealed that HIV-PIs are efficient drugs able to modulate vital pathophysiological processes in *F. pedrosoi*, as summarized in **Figure 7**. Thus, HIV-PIs may be candidates as lead compounds for the development of novel chromoblastomycosis treatments. Drugs with a distinct mechanism of action or a multitargeted combination therapy could be attractive to control *F. pedrosoi* infections. We previously showed that the combination of amphotericin B (3 μg/ml) and nelfinavir (25 μM), both at sub-inhibitory MIC values, caused significant killing (by around 70%) of *F. pedrosoi* conidial cells compared to the individual treatment with amphotericin B or nelfinavir. This finding indicated that both compounds acted synergistically to kill *F. pedrosoi* conidial cells (Palmeira et al., 2008). In this context, HIV-PIs could be used alone or in combination with current antifungal drugs leading to their use as alternative and topical therapeutic agents. It is important to emphasize that the creation of more-specific PIs possessing high, selective toxicity against *F. pedrosoi* and related fungi, would truly represent a therapeutic breakthrough in the fight against chromoblastomycosis.

## Author Contributions

VP, DA, LB-S, CA, SR, AS, and LK conceived and designed the experiments. VP, DA, LB-S, FG, and MG performed the experiments. All authors analyzed the data. DA, CA, SR, AS, and LK contributed reagents/materials/analysis tools. VP, DA, CA, SR, AS, and LK wrote and revised the paper. All authors contributed to the research and approved the final version of the manuscript. All authors agree to be accountable for all aspects of the work.

## Conflict of Interest Statement

The authors declare that the research was conducted in the absence of any commercial or financial relationships that could be construed as a potential conflict of interest.
